# Reliability of using a pressure sensor system to measure in-water force in young competitive swimmers

**DOI:** 10.3389/fbioe.2022.903753

**Published:** 2022-10-31

**Authors:** Catarina C. Santos, Daniel A. Marinho, Mário J. Costa

**Affiliations:** ^1^ Department of Sport Sciences, University of Beira Interior, Covilhã, Portugal; ^2^ Department of Sport Sciences, Polytechnic Institute of Guarda, Guarda, Portugal; ^3^ Research Center in Sports Sciences, Health Sciences and Human Development (CIDESD), Vila Real, Portugal; ^4^ Centre of Research, Education, Innovation and Intervention in Sport (CIFI2D), Faculty of Sport, University of Porto, Porto, Portugal; ^5^ Porto Biomechanics Laboratory (LABIOMEP-UP), University of Porto, Porto, Portugal

**Keywords:** swimming, kinetics, differential pressure, accuracy, hand force

## Abstract

The aim of this study was to analyze the reliability of using a differential pressure system to measure in-water force in young competitive swimmers. Ten boys and five girls (12.38 ± 0.48 years, 49.13 ± 6.82 kg, 159.71 ± 7.99 cm) were randomly assigned to perform two maximum bouts of 25 m front crawl on different days (trial one, T1; trial two, T2), one week apart. A differential pressure system composed of two hand sensors (Aquanex System, v.4.1, Model DU2, Type A, Swimming Technology Research, Richmond, VA, United States) was used to measure the peak (RF_PEAK_) and the mean (RF_MEAN_) resultant force of the dominant and non-dominant hands (in Newton, N). Reliability was analyzed by computing the intraclass correlation coefficient (ICC), typical error (TE), smallest worthwhile change (SWC), coefficient of variation (CV%), standard error of measurement (SEM), and the minimal detectable change (MDC). Bland–Altman plots with 95% limits of agreement were also analyzed. The results showed no differences between T1 and T2 in all variables (*p* > 0.05). The ICC showed “excellent” reliability (ICC > 0.90) for the RF_PEAK_ and RF_MEAN_ in both hands. The CV% was rated as “good” (<5%) and TE was smaller than SWC in all variables. The Bland-Altman plots showed high reliability with a small bias (RF_PEAK_ dominant, -0.29 N; RF_PEAK_ non-dominant, -0.83 N; RF_MEAN_ dominant, 0.03 N; RF_MEAN_ non-dominant, 0.50 N). The pressure sensor system (Aquanex System) seems to be a reliable device for measuring the hand resultant force during front crawl in young swimmers and can be used to monitor the changes over time.

## Introduction

Deterministic models of swimming performance have highlighted kinetics as an important domain to be studied (Barbosa T. M. et al., 2013). The ability of swimmers to move through the water depends on the amount of propulsive force applied and the drag force opposed to a forward motion. With that in mind, individual force profiles were used to understand propulsive mechanics in the water (Santos et al., 2021).

In the last couple of years, some progress has been made on how propulsive forces are retrieved (Santos et al., 2021). Methods with humans or robotic models based on numerical simulations (e.g., Marinho et al., 2010) or tethered swimming (e.g., Amaro et al., 2014) were used for that purpose; but those kind of approaches were quite heavy to handle or too much time consuming. Thus, the use of differential pressure sensors has been growing in interest. The method of assessing pressures differences between the palmar and dorsal surfaces, along with underwater motion analysis, allows to estimate the propulsive forces (Takagi and Wilson, 1999) and interpret those possible effects on performance (Tsunokawa et al., 2018; Koga et al., 2022). This straightforward method allows the assessment of swimmers in a more ecologically valid environment (i.e., similar to “free-swimming”).

Studies using the differential pressure method reported the measurement of in-water forces using two (e.g., Pereira et al., 2015; Bartolomeu et al., 2022) or four to eight sensors (e.g., Takagi and Wilson, 1999; Koga et al., 2020) in swimming strokes. Despite the number of sensors in play, the Aquanex System (a two-hand set-up) showed to be an easy-to-use procedure without encompassing a heavy set-up. This is an important advantage of the system when compared to other differential pressure sensors reported in the swimming science literature (e.g., Takagi and Wilson, 1999; Tsunokawa et al., 2018; Koga et al., 2022). Still, should point out that each sensor only measures the hand resultant force instead of the effective propulsive force. Although some studies reported the use of Aquanex System, the system accuracy and the reliability of the measurements has not yet been investigated. Meanwhile, young swimmers seem not to be constrained in stroke mechanics or stroke efficiency when using this system (Santos et al., 2022).

The peak and mean forces retrieved by this pressure sensors system have been regularly used to understand acute responses to different stimulus (e.g., Morais et al., 2020), the relationship to swimming velocities (e.g., Bartolomeu et al., 2022), upper-limb imbalances (e.g., Morais et al., 2020), or warm-up effects (e.g., Barbosa T. M. et al., 2020). Both kinetic variables appear to be highly reliable in young swimmers when using the tethered-swimming method (Amaro et al., 2014). However, it is still unclear whether the same happens when a pressure system with two hand sensors is used for this purpose. Thus, ensuring the reliability of the Aquanex System would help researchers and practitioners to perform a proper assessment over time and monitoring swimmers’ progress.

Thus, the aim of this study was to analyze the reliability of using a differential pressure system to measure in-water force during front crawl in young competitive swimmers. It was hypothesized that pressure sensors would present excellent reliability to measure the peak and the mean of hand resultant force.

## Materials and methods

### Participants

Fifteen highly trained (Mckay et al., 2022) swimmers including 10 boys and 5 girls [mean ± one standard deviation: 12.38 ± 0.48 years-old, 49.13 ± 6.82 kg, 159.71 ± 7.99 cm, 309.17 ± 58.13 FINA Points at 50-m freestyle (short course)] volunteered to participate in this study. Swimmers were recruited from a local swimming squad and assessed at the end of the first macrocycle (peak form). The inclusion criteria were defined as follows: 1) having a minimum of two years in competitive swimming in regional or national events; 2) practicing more than four swim training sessions per week; 3) being previously familiar with the hand differential pressure system; and 4) not having suffered any injuries in the past 6 months.

Swimmers’ parents or guardians were informed about the benefits and experimental risks before signing a written informed consent form. All procedures were in accordance with the Declaration of Helsinki and approved by the Institutional Ethics Committee of the University of Beira Interior (code: CE-UBI-Pj-2020-058).

### Data collection

A single group repeated measures design was selected for this study. The in-water experimental testing was carried out in a 25 m indoor swimming pool (water temperature: 27.5°C) and the swimmers attended two sessions on different days, 1 week apart. A standardized 1000 m warm-up for sprint events (Neiva et al., 2015) was performed individually by each swimmer. For the in-water data collection, swimmers were randomly assigned for the first maximum bout of 25 m front crawl (Trial 1, T1) and followed the same order in the second session (Trial 2, T2). All maximum bouts started by a push-off without gliding and swimmers were instructed to maintain their normal breathing pattern for sprint events.

Swimmers wore only a textile swimsuit and a cap during the anthropometric tests. Height (in cm) and body mass were measured with a digital stadiometer (SECA, 242, Hamburg, Germany) and a scale (TANITA, BC-730, Amsterdam, Netherlands), respectively. Hand dominance of the swimmers was assessed by self-report.

### Pressure sensors test

A differential pressure system composed of two hand sensors (Type A, Swimming Technology Research, Richmond, VA, United States) positioned between the third and fourth proximal phalanges and metacarpals was used to measure the pressure between the palmar and dorsal surfaces of both hands. Inside each sensor, there is a diaphragm that flexes and is sensed as an electrical signal that is proportional to the difference in the two pressures. Each sensor measures the pressure component acting perpendicular to it. The hand resultant force (in N) was derived by the system from the product of differential pressure by the hand surface area of each swimmer (i.e., differential pressure ∙ hand surface). The sensors (3.18 cm × 1.91 cm x 2.54 cm; 0.226 kg) were attached by a cable (15 m of length) to a two channel A/D interface connected to a laptop with the Aquanex software (v.4.1, Model DU2, Swimming Technology Research, Richmond, VA, United States). Swimmers carried the system with shoulders and arms elastic straps. An illustration of the experimental set-up can be found in Santos et al. (2022). Before each bout, swimmers kept their hands immersed (10 s) at the waistline to calibrate the system with the hydrostatic pressure values. Data was acquired with a sampling frequency of 100 Hz for each maximum bout.

### Data analysis

Data was imported into a signal-processing software (AcqKnowledge v.3.7.3, Biopac Systems, Santa Barbara, CA, United States) and the signal was handled with a 5 Hz cutoff low-pass fourth order Butterworth filter. The peak (RF_PEAK_, in N) and the mean (RF_MEAN_, in N) resultant force of the dominant and non-dominant hands were assessed during the underwater paths. The recovery phase was discarded for all cycles The RF_PEAK_ was defined as the maximum value achieved on the three consecutive stroke cycles analyzed between the 11^th^ and 24^th^ meter, as suggested elsewhere (Santos et al., 2022). The distance covered by the swimmers was recorded (Sony, HDR-CX 240, Japan) and a visual mark was applied in the defined interval. The RF_MEAN_ was defined as the mean of the values obtained from the force-time curve where the RF_PEAK_ was retrieved.

### Statistical analysis

The normality and homoscedasticity of the data were checked using the Shapiro-Wilk and Levene tests, respectively. The mean and one standard deviation (M ± 1SD) were computed as descriptive statistics. A paired sample *t*-test was used to compare the outcome variables between the T1 and T2. Relative test-retest reliability of each variable was assessed using the intraclass correlation coefficient (ICC) plus 95% confidence intervals (95%CI) with two-way mixed effects model (absolute agreement, single measures). The ICC was classified as poor if ICC < 0.50, moderate if 0.50 ≥ ICC < 0.75, good if 0.75 ≥ ICC < 0.90, and excellent if ICC > 0.90 (Koo and Li, 2016). The absolute test-retest reliability was analyzed by estimating the typical error (TE), coefficient of variation (CV%), standard error of measurement (SEM), and the minimal detectable change (MDC) based on a 95% confidence level (Atkinson and Nevill, 1998) The CV% values were interpreted as poor if CV% > 10%, moderate if 5% ≥ CV% ≤ 10%, and good if CV% < 5% (Scott et al., 2016). Additionally, the ability to detect a change was rated as “good”, “OK”, or “marginal” when the TE was below, similar, or higher than the smallest worthwhile change (SWC), respectively (Buchheit et al., 2011). Bland–Altman plots with 95% limits of agreement (LoA) were used to display the within-subject variation and systematic differences between the two sessions trials. The bias (mean difference), standard deviation (SD), and upper and lower LoA were calculated (Bland and Altman, 1986).

All statistical analyses were performed in the SPSS software (v.27, IBM, SPSS Inc., Chicago, IL, United States) and GraphPad Prism (v.9, GraphPad Software, San Diego, CA, United States). The statistical significance was set at *p* ≤ 0.05.

## Results

Test-retest reliability of the Aquanex System is shown in Table 1. No differences were found between T1 and T2 in all propulsive force variables. The ICC showed an “excellent” relative reliability for the RF_PEAK_ and RF_MEAN_ in both upper limbs, despite the 95%CI (i.e., lower and upper bound) of ICC demonstrating a “good” to “excellent” relative reliability. TE was rated as “good” when compared to the SWC and CV% revealed a “good” absolute reliability in all variables.

**TABLE 1:** Test-retest reliability of the Aquanex System in young competitive swimmers.

Variable	T1 (M ± 1SD)	T2 (M ± 1SD)	*p*-value	TE	SWC	CV%	ICC	ICC_95%CI_	SEM	MDC
RF_PEAK_ D (N)	50.02 ± 7.81	50.31 ± 8.29	0.65	0.96	1.61	2.70	0.96	0.88, 0.99	1.67	4.63
RF_PEAK_ ND (N)	49.85 ± 10.10	50.68 ± 9.87	0.17	0.99	1.99	2.95	0.97	0.92, 0.99	1.64	4.55
RF_MEAN_ D (N)	16.54 ± 3.49	16.51 ± 3.34	0.93	0.47	0.68	4.30	0.95	0.86, 0.98	0.75	2.07
RF_MEAN_ ND (N)	16.92 ± 3.44	16.42 ± 3.79	0.19	0.56	0.71	4.64	0.92	0.79, 0.97	1.00	2.76

D, dominant hand; CI, confident interval; CV%, coefficient of variation in percentage; ICC, intraclass correlation coefficient; ICC_95%CI_, lower and upper bound of ICC; MDC, minimal detectable change; N, newton; ND, non-dominant hand; RF_PEAK_, peak resultant force; RF_MEAN_, mean resultant force; SEM, standard error of measurement; SWC, smallest worthwhile change; T1, trial 1; T2, trial 2; TE, typical error.

**FIGURE 1:**
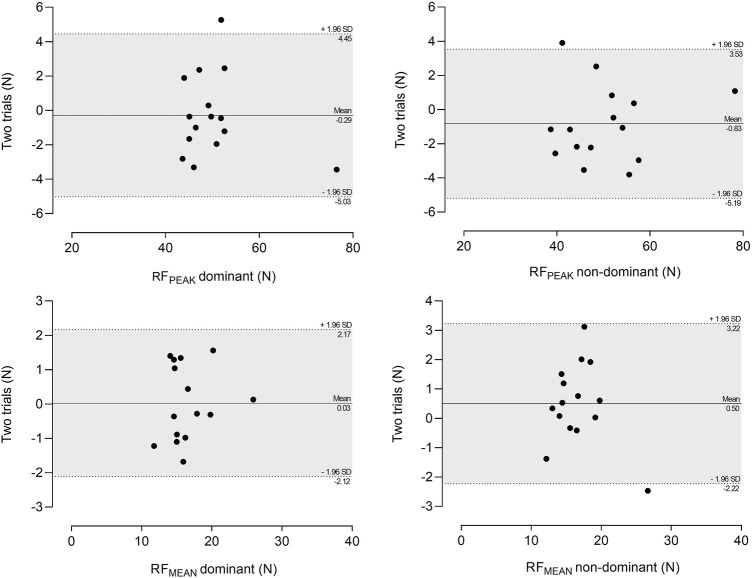
Bland-Altman plots of the difference between T1 and T2 (y-axis) and mean of measurements (x-axis) for all variables. Dotted lines represent the upper and lower 95% LoA (mean differences ± 1.96 SD of the differences) and solid lines represent the mean differences between the two trials (bias). N, newton; RF_PEAK_, peak resultant force; RF_MEAN_, mean resultant force.

The Bland-Altman plots are presented in Figure 1. Biases (mean differences) were small, approaching zero, and most data points were within the LoA on all resultant force variables.

## Discussion

This study analysed the reliability of using a differential pressure system to measure the hand resultant force during front crawl in young competitive swimmers. The main results show that the pressure sensor system has excellent reliability through the measurement of peak and mean resultant force.

Previous studies using the Aquanex system determined the peak and the mean as the most frequent variables to be studied (Santos et al., 2021). Our results showed values of ≈50 N for RF_PEAK_ and ≈17 N for RF_MEAN_. These values are lower than previous findings in front crawl stroke, but the age range reported was different from those used in the present study (e.g., Barbosa T. M. et al., 2020; Morais et al., 2020). Furthermore, studies reporting hand resultant force with multi-pressure system also found higher values (e.g., Tsunokawa et al., 2018; Koga et al., 2022).

The reliability of different devices/apparatus in swimming has been extensively investigated. Inertial measurement units (IMU) to assess in-water kinematics (Mooney et al., 2015) and dynamometers for dry-land strength assessment (e.g., Conceição et al., 2018) have already been tested. As far as we know, the reliability of devices to directly measure in-water forces have only been done using the tethered swimming method (Amaro et al., 2014; Nagle Zera et al., 2021). Hence, this study is the first to provide data about test-retest reliability with hand pressure sensors.

The ICC values observed in the present study were classified as “excellent” (range: 0.92–0.97) in both variables for the dominant and non-dominant hands. These results are in agreement with those observed in front-crawl in tethered swimming (Amaro et al., 2014; Barbosa A. C. et al., 2020; Dos Santos et al., 2017; Loturco et al., 2015; Morouço et al., 2014). For instance, Amaro et al. (2014) reported high reliability for peak (ICC: 0.94) and mean forces (ICC: 0.96) in young swimmers. Although tethered swimming is considered a reliable apparatus, some concerns have been raised as swimmers remain in stationary conditions with no forward motion (Soncin et al., 2017). Furthermore, it is expected that with such method swimmer’s hand would experience much larger pressure than in a free-swimming condition. On the other hand, the pressure sensors allow a displacement throughout the water without mechanical and efficiency constraints in young swimmers (Santos et al., 2022).

Although the reliability of the two pressure sensors has not been investigated in previous studies, Havriluk (1988), who introduced the first version of the Aquanex System, reported an ICC value of 0.91 for the variable “effective hand movement with respect to the body” (in m). Nevertheless, in-water force values were not analyzed, therefore, no conclusions were drawn about reliability.

The absolute reliability demonstrated a “good” CV% without systematic changes between trials. The CV% ranged from 2.70 to 4.64% and the TE was below 1N being rated as “good” when compared to the SWC. The SEM was less than 2N in all variables. Thus, the differential pressure system (Aquanex System) might be a reliable apparatus to monitor changes in hand resultant force over the season. Meanwhile, different CV% values have been reported for tethered swimming, being lower (Barbosa A. C. et al., 2020; Loturco et al., 2015) or higher (Amaro et al., 2014) than those found in the present study. Different settings, such as the competitive level of the sample, swimmers’ age, or data analysis, can help explain these differences.

Some limitations can be addressed: 1) equal pressure assumption on the hand surface, although it has been shown that the pressure is not the same across the whole surface of the hand; 2) only the resultant force was considered; 3) only the reliability of the hands was considered, although the in-water forces of the feet’s has also been investigated through pressure sensors. Thus, testing its reliability alone or using the set-up of the hand should be a priority in the future; 4) only peak and mean forces of young swimmers were considered; the use of other measures (e.g., impulse) and type of swimmers (e.g., elite or master) would be essential; and 5) front-crawl is not representative of all swimming strokes, so future studies should try to understand whether systematic changes are the same for butterfly, backstroke, and breaststroke.

### Practical applications

Defining the most important factors of swimming performance within the biomechanical domain is still a challenge. This is mainly due to the aquatic environment, which is the biggest obstacle to be overcome in the search for more accurate assessments. Within this rationale, new technologies, such as pressure sensors, make a great contribution to this area. The potentiality of monitoring the hand force continuously during the free-swimming without spatial limitations is an advantage of the Aquanex system with two sensors. As far as we know, this is not possible with other methods that assume the swimmer’s kinetic variables. However, the accuracy of such system has not been previously demonstrated. Furthermore, it would be appreciable as a future perspective the wirelesses of these sensors (i.e., telemetry). So, this study is the first to show the reliability of using the Aquanex system for measurements of peak and mean forces in water. This will allow for a deeper understanding of how swimmers generate in-water forces and it will help coaches redefining training programs at some point of the competitive season, if necessary. For researchers, the link between hand forces retrieved by Aquanex and the remaining determinant domains of performance (e.g., anthropometric, biomechanical, physiological) should be a focus in a near future.

## Conclusion

The pressure sensor system (Aquanex System) can be considered a reliable set-up to obtain peak and mean hand resultant force in young competitive swimmers. This reinforces the idea that the use of pressure sensors remains the assessment method that most closely resembles free-swimming and can be used to monitor kinetic changes over time.

## Data Availability

The raw data supporting the conclusions of this article will be made available by the authors, without undue reservation.
